# Ocular Comorbidities and the Relationship between Eye Diseases and Systemic Disorders

**DOI:** 10.1155/2016/9519350

**Published:** 2016-03-29

**Authors:** Maria D. Pinazo-Durán, J. Fernando Arévalo, José J. García-Medina, Vicente Zanón-Moreno, Roberto Gallego-Pinazo, Carlo Nucci

**Affiliations:** ^1^Ophthalmic Research Unit “Santiago Grisolía”, Fundación Investigación Biomédica y Sanitaria (FISABIO), 90 Gaspar Aguilar Avenida, 46017 Valencia, Spain; ^2^Ophthalmology Research Unit, Department of Surgery, Faculty of Medicine and Odontology, University of Valencia, 15 Blasco Ibañez Avenida, 46010 Valencia, Spain; ^3^Spanish Net of Ophthalmic Pathology OFTARED, Institute of Health Carlos III, 4 Sinesio Delgado Street, 28029 Madrid, Spain; ^4^Retina Division, The Wilmer Eye Institute, The Johns Hopkins University School of Medicine, Baltimore, MD, USA; ^5^Ophthalmology Department, University Hospital Reina Sofía, 1 Intendente Jorge Palacios Avenida, 30003 Murcia, Spain; ^6^Department of Surgery, Faculty of Medicine, University of Murcia, Campus Universitario de Espinardo, s/n, Espinardo, 30100 Murcia, Spain; ^7^Ophthalmology Department, University and Polytechnic Hospital “La Fe”, 106 Fernando Abril Martorell Avenida, 46026 Valencia, Spain; ^8^Ophthalmic Unit, Department of Experimental Medicine and Surgery, University of Rome Tor Vergata, Rome, Italy

Patients with ocular disorders may have additional ophthalmic problems that can have an impact on both morbidity and vision. Ocular comorbidities are commonly associated with vision-related disability and decreased quality of life related to visual impairment. The majority of studies on this topic deal with cataracts, glaucoma, uveitis, and/or retinopathies. It is important to summarize the available evidence to date on the association of one or several ocular diseases and the implications these comorbidities have on prognosis and therapy.

The relationship between eye disorders and systemic diseases has recently drawn special interest. The increasing prevalence of neurodegenerative disorders, diabetes mellitus, hypertension and cardiovascular pathologies, and osteoarticular processes has a great impact on the society. In order to appropriately manage eye disorders, it is pivotal to achieve an early and accurate diagnosis of concomitant systemic diseases. This type of integrated approach is essential to ensure a better knowledge of the comorbidities to prevent blindness.

In this issue, a variety of works have precisely addressed important ocular comorbidities, as well as the state of the art on the relationship between eye diseases and systemic disorders. A total of 6 reviews, 3 clinical studies, and 4 research articles have been joined herein to nicely draw the multidisciplinary scenario of this special issue.

Regarding the review articles, M. Figus et al. go over Adamantiades-Behçet's disease with the most serious clinical manifestation being bilateral panuveitis, which may lead to blindness. The authors show that the use of biological agents improves the visual prognosis of the affected patients. H. J. Chung et al. take a close look at the relationship between primary open-angle glaucoma (POAG) and blood pressure (BP), showing that the increase in the latter induced an increase of the intraocular pressure (IOP) leading to a higher risk of glaucoma progression. E. M. Vingolo et al. provided a complete guide to reduce the risk of ocular manifestations due to Ebola virus disease. F. J. Muñoz-Negrete et al. reviewed current knowledge on the diagnosis and management of uveitic glaucoma. Since this type of glaucoma is related to very high IOP and more severe optic nerve damage than other types of glaucoma, it is mandatory to diagnose as soon as possible this comorbidity and the most appropriate management of both uveitis and glaucoma. A. C. Martins et al. revised the ocular manifestations associated with the familial amyloid polyneuropathy, with the amyloid deposition in the vitreous, dry eye, and secondary glaucoma being the most relevant. Further investigations are needed in this regard to develop new therapeutic strategies to avoid or treat these ocular disorders. Finally, M. D. Pinazo-Durán et al. had a quick look at selected ocular comorbidities, such as dry eyes, glaucoma, cataracts, and retinopathies, as well as the eye manifestations in systemic diseases with special attention to the aging eyes.

The clinical studies deal with interesting subjects. H.-C. Kau and C.-C. Tsai assessed the clinical features of nasopharyngeal carcinoma patients with new onset diplopia after concurrent chemoradiotherapy and they found that this kind of diplopia could be caused by tumor recurrence or treatment complications with different manifestations or prognosis. J. L. Alio et al. compared the results of femtosecond laser versus manual technique for deep anterior lamellar keratoplasty and they concluded that both methods had similar visual and refractive outcomes with more evident wound healing when using laser. Finally, A. Ribelles et al. investigated the influence of working with computers on ocular surface in a sample of older women. These authors also studied the effect of oral supplementation with antioxidants/omega 3 fatty acids on ocular surface features in this context. They demonstrated that computer use during the working time induced obvious ocular surface sign and symptoms. Otherwise, supplementation was demonstrated to positively ameliorate this situation.

Four research articles have addressed the role of ocular comorbidities and the relationship between the systemic disorders and eye diseases. F. J. Muñoz-Negrete et al. showed the results of a study for implementing the diagnosis of glaucoma in diabetics by means of a set of criteria based on nonmydriatic monoscopic fundus photography. E. Salobrar-Garcia et al. performed screening of the peripapillary and macular segmentation thickness by optical coherence tomography in patients with Alzheimer's disease as compared to age-matched control subjects. The authors concluded that the increase in peripapillary thickness in patients affected with mild disease could correspond to an early neurodegeneration stage closely related to inflammation. J.-C. Yen et al. have done a nationwide population-based study on the risk factors of retinal artery occlusion, concluding that atrial fibrillation and coronary artery disease are the most relevant causes that may induce retinal vascular alterations. M. J. Roig-Revert et al., on behalf of the Valencia Study Group of Diabetic Retinopathy (VSDR), presented a research article on the biomarkers for diabetic retinopathy. Data from this study showed that plasmatic oxidative stress biomarkers were significantly higher in diabetics than in the control subjects. This status significantly improved in the subgroup of participants daily taking oral supplements with antioxidants and omega 3 fatty acids during 18 months of follow-up.

To summarize, this collection of papers covering different topics can be useful for medical specialists and interdisciplinary researchers to improve our understanding of the mechanisms underlying the ocular comorbidities as well as the relationship between eye diseases and systemic disorders.

Being the editors of this special issue, we hope that the readers can appreciate all these works that may also contribute to moving this important topic forward by stimulating innovative diagnostic and therapeutic strategies for better eye care.

*Maria D. Pinazo-Durán* *J. Fernando Arévalo* *José J. García-Medina* *Vicente Zanón-Moreno* *Roberto Gallego-Pinazo* *Carlo Nucci*

